# Left Atrial Appendage: Physiology, Pathology, and Role as a Therapeutic Target

**DOI:** 10.1155/2015/205013

**Published:** 2015-07-07

**Authors:** Damiano Regazzoli, Francesco Ancona, Nicola Trevisi, Fabrizio Guarracini, Andrea Radinovic, Michele Oppizzi, Eustachio Agricola, Alessandra Marzi, Nicoleta Carmen Sora, Paolo Della Bella, Patrizio Mazzone

**Affiliations:** ^1^Non-Invasive Cardiology, Cardio-Thoracic-Vascular Department, San Raffaele Scientific Institute, Via Olgettina 60, 20132 Milano, Italy; ^2^Arrhythmology and Electrophysiology Unit, Cardio-Thoracic-Vascular Department, San Raffaele Scientific Institute, Via Olgettina 60, 20132 Milano, Italy

## Abstract

Atrial fibrillation (AF) is the most common clinically relevant cardiac arrhythmia. AF poses patients at increased risk of thromboembolism, in particular ischemic stroke. The CHADS2 and CHA2DS2-VASc scores are useful in the assessment of thromboembolic risk in nonvalvular AF and are utilized in decision-making about treatment with oral anticoagulation (OAC). However, OAC is underutilized due to poor patient compliance and contraindications, especially major bleedings. The Virchow triad synthesizes the pathogenesis of thrombogenesis in AF: endocardial dysfunction, abnormal blood stasis, and altered hemostasis. This is especially prominent in the left atrial appendage (LAA), where the low flow reaches its minimum. The LAA is the remnant of the embryonic left atrium, with a complex and variable morphology predisposing to stasis, especially during AF. In patients with nonvalvular AF, 90% of thrombi are located in the LAA. So, left atrial appendage occlusion could be an interesting and effective procedure in thromboembolism prevention in AF. After exclusion of LAA as an embolic source, the remaining risk of thromboembolism does not longer justify the use of oral anticoagulants. Various surgical and catheter-based methods have been developed to exclude the LAA. This paper reviews the physiological and pathophysiological role of the LAA and catheter-based methods of LAA exclusion.

## 1. Introduction

The Left atrial appendage (LAA) has a complex anatomical structure that is distinct from the rest of the left atrium as it has different embryologic, anatomic, and pathophysiologic characteristics.

LAA is a remnant of the embryonic left atrium [1], while the rest of the left atrial cavity derives from an outgrowth of the pulmonary veins.

In order to define LAA anatomy and topographic relationships, multidetector computerized tomography (CT), and its high definition and transesophageal echocardiogram (TEE), in particular with the development of three-dimensional reconstructions, are the most accurate noninvasive imaging modalities. Cardiac magnetic resonance imaging (MRI) is an emerging technique in order to detect thrombi as well as LAA sizing [2], but its use in the clinical setting remains limited mainly due to its high costs and poor temporal resolution.

LAA is not just an embryologic remnant, but it seems to play an important role in the regulation of heart rate and fluid balance [3].

On the other hand, LAA has a key role in the thromboembolic risk [4] associated with atrial fibrillation (AF) and it could also have a possible triggering effect of atrial tachyarrhythmias [5].

Because of this role in physiology and pathophysiology, LAA is recently gaining attention as a therapeutic target especially in thromboembolism prevention in patients with AF.

In fact, it is the most common source of cardioembolic stroke in AF as LAA thrombus is present in up to 15% of patients in AF [6] and 90% of thrombus formation in nonvalvular AF is in LAA [7].

For this reason it has been defined as the “most lethal human appendage” [8] causing significant mortality and morbidity in AF patients.

CHADS2 score (cardiac failure, hypertension, age, diabetes, diabetes, and stroke) and the more recent CHA2DS2-VASc score (with the addition of gender, vascular disease) are useful tools to stratify thromboembolic risk in AF patients, guiding the decision for anticoagulation therapy [9, 10]. In fact, oral anticoagulation (OAC) has been shown to significantly reduce the risk of thromboembolism in numerous studies [11].

However, due to poor patient compliance, contraindications, and potential bleeding complications, OAC is underutilized in AF [12].

So, in certain clinical scenarios, when anticoagulation is contraindicated or has a high risk, LAA percutaneous closure is a safe and effective measure to prevent thromboembolism.

Considering that only 10% of the clinically relevant emboli in nonvalvular AF do not originate in the LAA [13], with the exclusion of LAA as an embolic source, the remaining small risk does not require any longer OAC with its inherent risk for side effects, especially major bleedings. Numerous methods have been developed to exclude the LAA, surgically or percutaneously, LAA [14, 15].

## 2. Physiology and Pathophysiology of the LAA

### 2.1. Anatomy and Physiology

**Figure 1:**
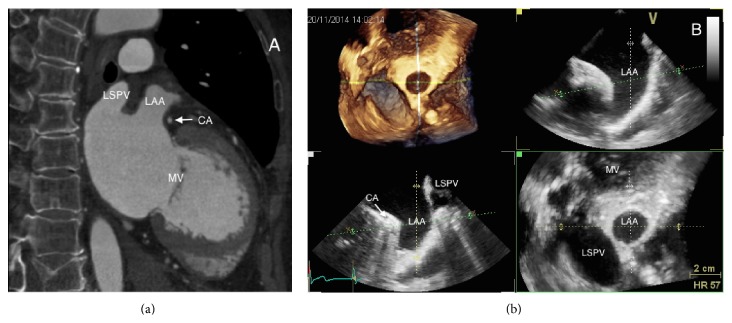
Left appendage anatomical relationship, as seen with cardiac CT scan (a) and 3D transesophageal echocardiography (b). LAA: left atrial appendage; LSPV: left superior pulmonary vein; CA: circumflex artery; MV: mitral valve.

The LAA is a remnant of the embryonic left atrium [1], lying in the left atrioventricular groove and in close relation with the left circumflex artery, with the left superior pulmonary vein posteriorly, with the mitral valve annulus medially, and with the left phrenic nerve laterally (Figure 1).

Anatomical studies have described numerous shapes of the LAA: as a long, narrow, tubular, and hooked structure [17].

The shape of the LAA ostium is typically elliptical (68.9%), with a long diameter ranging from 10 to 40 mm and a maximal depth ranging from 16 to 51 mm [18]. A round shape is present only in 5.7% of cases. Interestingly, ostium diameters showed minimal changes during different phases of the cardiac cycle in sinus rhythm (maximal change 1 to 2 mm), while no change was observed during AF [19].

Veinot et al. [20] have examined 500 anatomical findings: in more than two-thirds of cases, LAA is composed of two or more lobes, located in different planes. Classically, the lobes head toward the atrioventricular groove and the basal surface of the left ventricle. This has to be kept in mind during imaging studies in order to rule out intracavitary thrombus: failure to view all the lobes or incomplete visualization of a lobe may account for underdiagnosis of LAA thrombosis.

Recently, a CT based study classified LAA morphology on the basis of the presence of a bend, giving to the LAA an appearance similar to a chicken wing (48% of cases). Others possible morphologies are cactus shape (30%), with a dominant central lobe and secondary lobes extending from the central lobe in both superior and inferior directions; windsock shape (19%), with 1 dominant lobe; cauliflower shape (3%), with limited overall length and complex internal characteristics [21].

Histologically, the LAA has a single layer of endothelium and contains pectinate muscles with variable thickness [19]. The anterolateral wall, close to the mitral valve, has the minimum thickness (0.5 mm): particular care should be taken to avoid perforation during invasive procedures.

As said before, the LAA does not seem to be just an embryologic remnant, a useless appendage. The LAA is responsible for several functions: it acts as a reservoir during left ventricular systole, a conduit for blood transiting from the pulmonary veins to the left ventricle during early diastole, an active contractile chamber that augments left ventricular filling in late diastole, and a suction source that refills itself in early systole [22]. In fact, the LAA seems more distensible than the rest of the atrium and it could act as a volume reserve. Experimental findings during cardiac surgery demonstrated how LAA temporary exclusion augments LA pressure [23]. It is also possible that the LAA could contribute to stroke volume, due to its intrinsic contractile capability [24].

The LAA also has an endocrine role: it contains stretch-sensitive receptors that are able to influence heart rate and natriuretic peptides secretion in response to change in atrial pressure. A quantitative analysis of atrial natriuretic peptides (ANP) in excised LAAs revealed a content of approximately 30% of all cardiac ANP [25]. Experimental infusion of fluid in the LAA results in diuresis and natriuresis and increased heart rate, supporting a significant role of the LAA in regulating normal cardiac physiology [26].

However, little is known about these functions in a pathological LAA, as seen during AF or after LAA closure.

**Figure 2:**
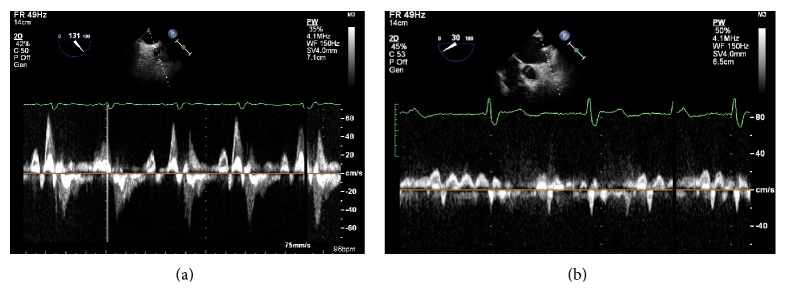
Left atrial appendage emptying velocity during normal sinus rhythm (a) and during atrial fibrillation (b).

The LAA has a distinct pattern of contraction, extensively studied with TEE [27]. It has an augmented contractility in respect to the atrium; typically, it has a biphasic pattern of emptying, a first passive phase in protodiastole and a second active phase during left atrial contraction and a prominent monophasic pattern of filling (Figure 2(a)). During atrial fibrillation, the pattern is characterized by a rapid alternation of emptying and filling, with lower velocities (Figure 2(b)).

Abnormalities of the LAA function observed at TEE in AF (perturbations of LAA emptying peak flow velocity, LAA fractional area change and LAA velocities <0.2 m/sec), are associated with the occurrence of spontaneous echo-contrast and thrombus formation resulting from blood stagnation in the LA. These findings have been shown to be associated with the occurrence of ischemic strokes in several clinical reports [28].

### 2.2. Role of the LAA in Cardiac Pathophysiology

As mentioned before, the LAA is the most common source of cardioembolic stroke in nonvalvular AF (up to 90% of cases) [7].

The reasons why this happens are multiple, summed up by the Virchow triad [29]. First of all, the risk of thrombus formation depends on the hemodynamic function of the LAA. Three LAA flow patterns have been described:

1. type I, characterized by a regular biphasic emptying pattern, occurring in sinus rhythm;
2. type II, characterized by a saw-tooth emptying pattern, occurring in some patients with atrial fibrillation;
3. type III, without any active emptying pattern, typically occurring during AF. This is associated with the highest incidence of spontaneous echo-contrast and thrombus [30].

A reduced LAA peak flow velocity is considered as one of the strongest independent predictors of an increased thromboembolic risk [31].

Furthermore a prothrombotic and hypercoagulable state in AF has been demonstrated, manifested by increased blood levels of markers, reflecting coagulation activity (e.g., prothrombin fragments 1 and 2, fibrinopeptide A, thrombin–antithrombin complexes, and D dimer) [32].

Eventually, atrial fibrillation leads to damages, fibrosis, and inflammation of the endothelium of the left atrium, especially of the LAA [33].

In addition to these factors, LAA shape and size have also been recently evaluated as additive risk factors: in fact spontaneous echo-contrast is most likely found in larger LAA with more complex anatomies [21].

## 3. LAA as a Target for Thromboembolic Risk Prevention

Although antiarrhythmic drugs and catheter ablation are effective in symptomatic relief for patients with atrial fibrillation, the prevention of thromboembolic events is still entrusted to oral anticoagulation (vitamin K antagonists, VKA), irrespective of the rhythm management strategy.

With the recent emergence of new antithrombotic drugs (i.e., dabigatran, rivaroxaban, apixaban, and edoxaban), it became clear that VKA, although being more effective than aspirin and combination aspirin-clopidogrel, is often not well tolerated by patients, has a very narrow therapeutic range, and has a high risk for bleeding complications. However, the drugs mentioned above do not completely solve the risk of bleeding related to antithrombotic therapy.

This is why, over the years, several clinical trials have assessed the feasibility and efficacy of LAA occlusion as a tool for thromboembolism prevention in AF. The first interventions were performed by surgical ligation or removal of the LAA during valvular operations. In fact, LAA obliteration was first suggested as an addition to mitral valvotomy, even before the advent of cardiopulmonary bypass [4]. About fifty years later, interest in surgical LAA exclusion increased after the development of Maze procedure, performed by Cox, that was a reliable solution for the treatment of AF and included atrial appendage removal [34]. Since then, the procedure has evolved in two directions: LAA exclusion with sutures on the epicardial or endocardial surface and LAA excision through staples or removal and oversew. The surgical literature on LAA closure consists primarily of retrospective case series and, regardless of the approach used, showed that incomplete LAA closure may be worse than no closure [35]. All available studies show a failure rate between 10% [36] and 55% [37] with the consequent effect of potentially increasing the incidence of stroke.

**Figure 3:**
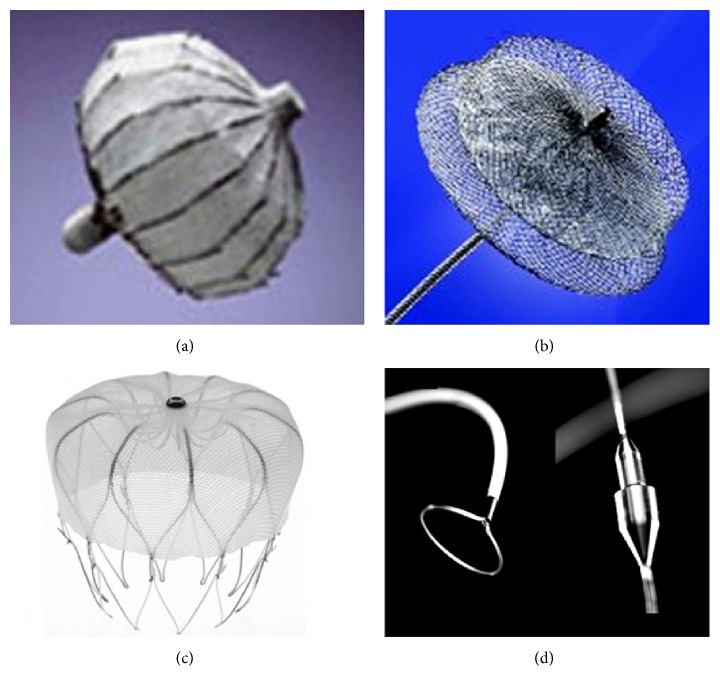
Left atrial appendage occlusion devices: PLAATO device, no longer available (a), Amplatzer Cardiac Plug (b), Watchman (c), and Lariat device (d).

The vast majority of patients, however, suffers from nonvalvular atrial fibrillation and has no indications for cardiac surgery: this is why in the last decade percutaneous approaches for LAA occlusion were developed. Obstruction of the LAA orifice with an occlusion device [38] or percutaneous suture ligation using an endocardial/epicardial approach [39] is the two alternatives (Figure 3).

The first percutaneous LAA occlusion was performed by the electrophysiologist Michael Lesh with a device called PLAATO (Percutaneous Left Atrial Appendage Transcatheter Occluder) on 30 August 2001 [40].

After that, several studies using the PLAATO device have been published: in the international multicenter feasibility trials [41], device implantation was successful in 108 of 111 patients (97.3%) with only one cardiac tamponade and one major vascular complication. The postprocedural stroke incidence was lower than that projected by the clinical scores (mean CHADS2 score 2.5). In fact, the incidence of the major and minor stroke was under 2% during a mean follow-up period of about one year.

Further studies showed that the PLAATO device appeared to be effective in reducing the stroke risk in patients with AF (stroke incidence 2.3%/year versus 6.6%/year as predicted by CHADS2 score), with a small risk of major periprocedural events (procedural success 90%, cardiac tamponade 3.3%, acute mortality 1.1%, and device embolization 0.6%) [42].

However, the PLAATO device has been discontinued for commercial reasons and then it was withdrawn from the market.

The Amplatzer Cardiac Plug (ACP) has the longest clinical follow-up among the currently available LAA occluders [43].

Trials have shown that complications, such as pericardial effusion leading to cardiac tamponade, occurred in 2% of patients, as did the neurological events (Table 1).

Technical success was 97% and a relevant thrombus on the device during follow-up TEE was seen in 3%. The percentage of residual peridevice flow is very low: at 6 months, TEE is about 1%, probably due to the device's peculiar shape, with a disk that occludes the so-called “mouth” of the LAA. Generally, antithrombotic therapy after ACPs implant relies on double antiplatelet therapy instead of OAC.

The second generation of Amplatzer device, the so-called Amulet device, has recently been introduced in the market: Amulet data are available in only a few patients, although it was used in over 250 patients in Europe. The device [44, 45] has new features as compared to the first generation ACP as it shows good deliverability (96%–100% of procedural success) with 0%–5% pericardial effusion incidence and without acute strokes or device embolization.

The Watchman device is the only one evaluated in prospective, controlled, randomized trials (Table 2) examining its efficacy and safety (PROTECT AF trial [38] and PREVAIL trial [46]).

In the PROTECT AF, that enrolled 707 patients for 1065 patient-years of follow-up, a relative risk reduction of 46% of ischemic strokes and systemic thromboembolism (from 2.85 to 1.53) was observed in comparison to the control group, although a higher rate of adverse safety events was noted, mainly due to periprocedural complications such as pericardial effusion and procedural stroke typically related to air embolism.

Also the PREVAIL study, in patients with higher risk, demonstrated low-early and long-term primary and safety event rates.

Furthermore, a cost-efficacy analysis was carried out and showed that LAA occlusion was cost-effective when compared to warfarin and dabigatran (but only marginally with the latter drug) [47].

A subanalysis of patients enrolled in PROTECT AF showed that residual peridevice flow is possible after device implantation. However, small peridevice residual flow does not seem to have an impact on safety and clinical efficacy of Watchman implantation [48].

The WaveCrest device has recently received a CE mark as well, and it seems to provide a more superficial deployment with little or no manipulation within the LAA body. The WaveCrest device consists of a nitinol structure without exposed metal hub and with a foam layer facing the LAA to promote rapid organization and a PTFE layer facing the LA to reduce thrombus formation. Procedural and follow-up data are not available yet for this device.

The Lariat combined endocardial/epicardial suture ligation of the LAA uses a combination of transseptal placement of a temporary balloon in the LAA, magnet-tipped guidewires inserted into the LAA and the pericardial space, and a closure snare device. This device demonstrated successful LAA closure in a canine model [49]. Studies in the human population [50, 51] in almost 200 patients showed a good procedural success (93-94%). Early major complications were higher as compared to fully percutaneous devices: the incidence of pericardial effusion requiring pericardiocentesis was between 11% and 20%, and the incidence of major bleeding was 9% while another 9% of patients suffered LAA perforation needing open chest surgery. At the follow-up incidence of stroke, myocardial infarction was under 3%/year. Furthermore, LAA exclusion with this device appears to reduce AF burden [52], thus confirming the role of the LAA in triggering AF.

## 4. Imaging for Left Atrial Appendage Occlusion

Adequate imaging modalities are essential for successful LAA occlusion, by guiding preprocedural planning and periprocedural assessment and follow-up.

This usually requires TEE or CT. TEE is crucial in guiding the procedure of LAA occlusions [53], and it is recognized as the gold standard for it.

### 4.1. Preprocedural Assessment

At first, it is important to confirm the absence of thrombi in LAA before the procedure, in order to avoid a possible embolization with sheath or device manipulation.

**Figure 4:**
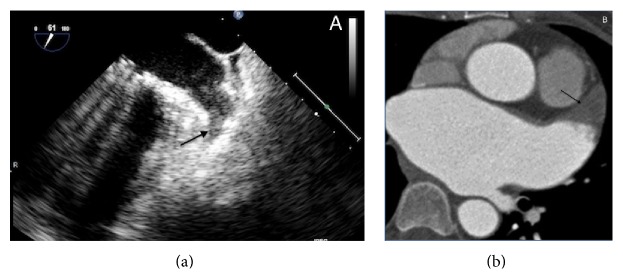
Left atrial appendage thrombosis (arrow), as seen with transesophageal echocardiography (a) and CT scan (b).

**Figure 5:**
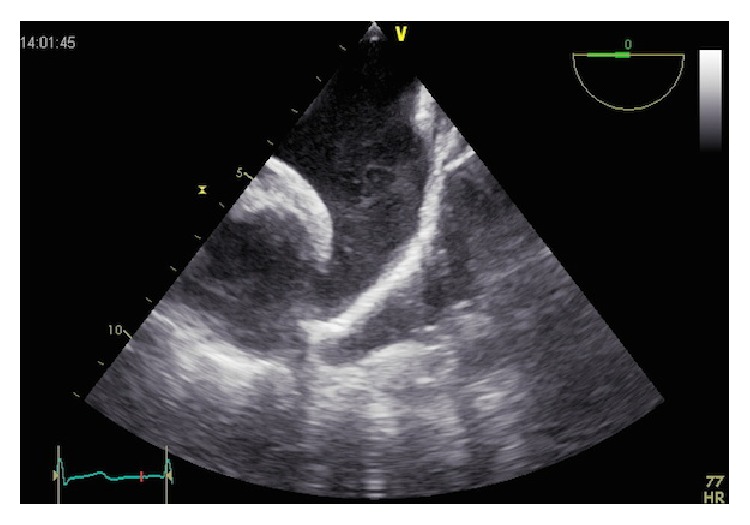
Severe spontaneous smoke effect (sludge) in left atrial appendage.

The imaging technique that is more validated and more often used for this purpose is TEE: in some patients, there may still be difficulties in differentiating prominent pectinate muscles from LAA thrombi. However, the incidence of LAA thrombus (Figure 4(a)) or sludge (Figure 5) among patients undergoing AF ablation who have been adequately anticoagulated was found to be very low, and it is well correlated with the CHA2DS2-VASc score [54].

Dual-enhanced cardiac CT [55] and cardiac MRI [56] could also be useful for this purpose (Figure 4(b)).

Spontaneous echocardiographic contrast is diagnosed in the presence of smoke-like echoes with a characteristic swirling motion, when the gain settings have been increased in a stepwise manner.

A thrombus is diagnosed if an echo reflecting mass is evident in more than one imaging plane, with independent mobility.

If a thrombus is detected inside the LAA, it is prudent to optimize anticoagulation and reassess LAA status after 4 weeks of optimal anticoagulation therapy.

In case of persistence, it is possible to surgically remove the thrombus and exclude the LAA. Percutaneous procedures have also been performed in this setting with an embolic protection device in the supra-aortic trunks [57].

However, it seems prudent not to perform this procedure in case of LAA thrombosis.

**Figure 6:**
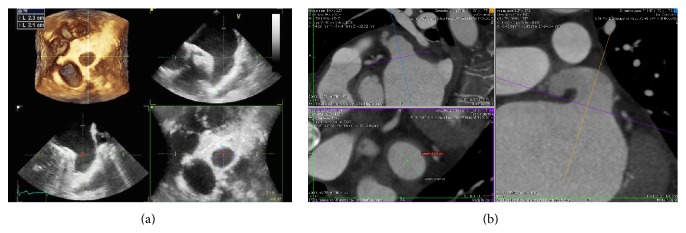
Left atrial appendage measures with transesophageal echocardiography (a) and CT scan (b).

Preprocedural TEE guides the decision of the device size: multiplane views (midesophageal 0°, 45°, 90°, and 135°) characterize LAA shape and morphology, facilitated by 3-dimensional reconstructions (Figure 6(a)).

It has to be kept in mind that 2D TEE underestimates true dimensions in comparison to 3D TEE or CT measurements (Figure 6(b)) [58].

The maximal width of the LAA ostium is measured from the level of the left circumflex coronary artery up to a point at 1-2 cm from the tip of the left superior pulmonary vein limbus.

The maximal depth is measured from the ostium line to the apex of the LAA. Sizing tables are available for both the Watchman and ACP devices.

The size of the chosen device should at least be 10–20% larger than the measured diameter: a correct oversizing is essential in order to avoid peridevice flow after deployment; on the other side, excessive oversizing may result in a compression of the left circumflex artery.

It is worth noting that the ostium of the LAA is typically elliptical, while all available occluders have a round shape, possibly accounting for incomplete sealing of the device and possible cause of leakages.

ACP has to be preferred if the depth of the LAA is smaller than the width of the ostium, because the placement of a Watchman device may result in an unstable position.

Preprocedural TEE evaluation could also be useful to better assess the thromboembolic risk of the patient. LAA dimensions, LAA velocities, left atrial dimensions and fibrosis, left ventricular dysfunction, spontaneous echo-contrast, and aortic plaque (especially in aortic arc) have been associated with an increased thromboembolic risk [59]. In particular, the presence of left atrial abnormalities is associated with an embolic risk of 7.8%/year, as well as a CHA2DS2-VASc score of 5.

All these data could be useful to guide decisions on thromboembolic risk prevention in case of borderline CHA2DS2-VASc scores.

### 4.2. Procedural Imaging to Guide Left Atrial Appendage Occlusion

TEE allows us to visualize the LAA during the procedure: it is essential for the positioning and deployment of the device.

The majority of centers perform the procedure under general anesthesia with TEE and fluoroscopic guidance. There are only few reports of intracardiac echocardiographic guidance during percutaneous LAA occlusion.

**Figure 7:**
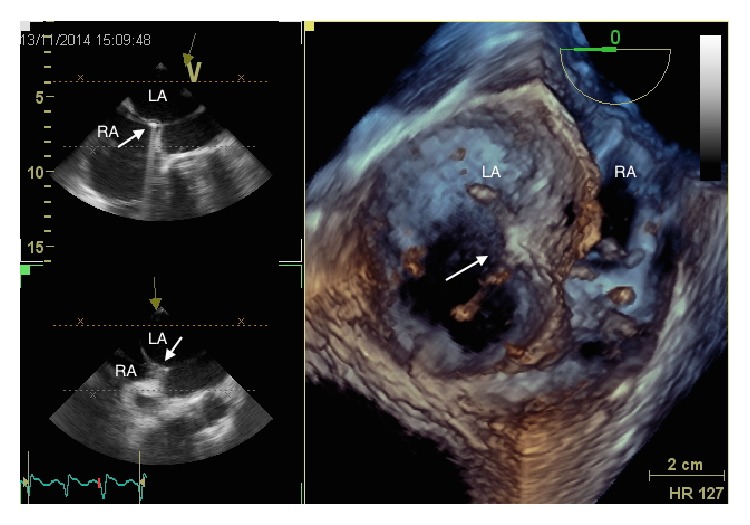
Real-time 3D echocardiography during transseptal puncture. The tip of the catheter (arrow) is passing from the right atrium (RA) to the left atrium (LA), through interatrial septum.

**Figure 8:**
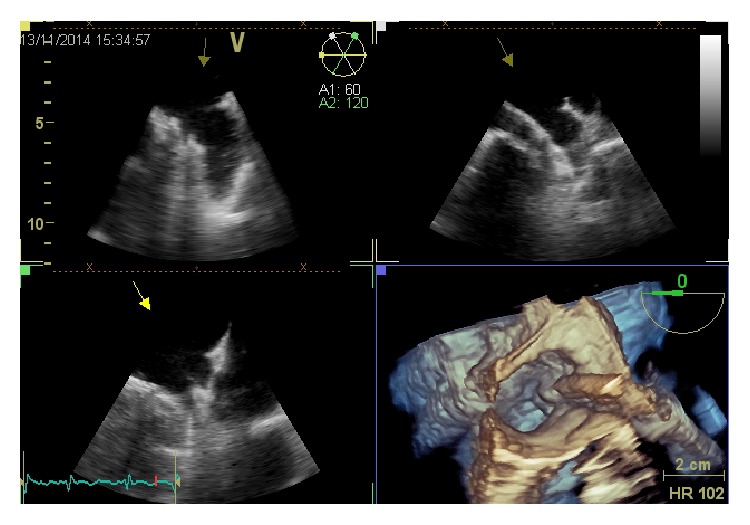
Progress of the delivery system in the left atrial appendage.

TEE facilitates the transseptal puncture (Figure 7) and especially 3D TEE can provide a real-time full view of the LAA, the shape of the ostium (Figure 8), with accurate measurements of the landing zone.

A final decision on device size is based on the information collected with all imaging modalities: echocardiography, fluoroscopy, and CT.

**Figure 9:**
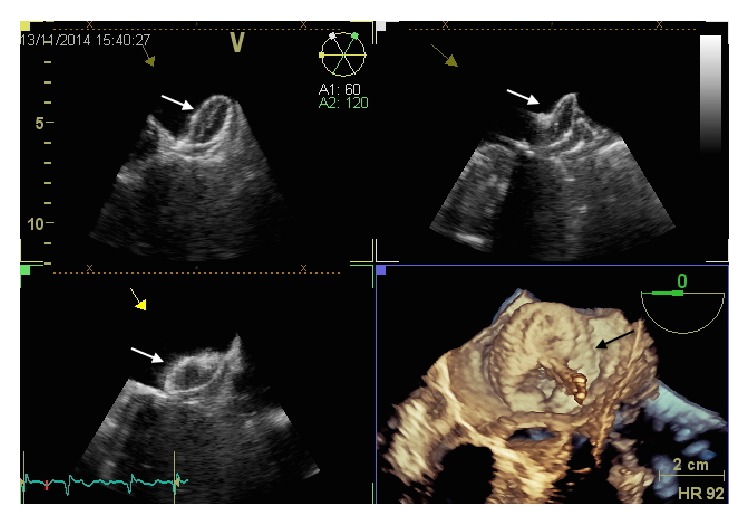
The image shows the so-called “tug test.” An Amplatzer Cardiac Plug is pulled before the deployment. During this maneuver, the distal part of the device (“disk,” arrow) is put in tension, while the distal part (“lobe”) remains anchored in left atrial appendage.

After deployment, a tug test should be performed demonstrating simultaneous movement of the device and appendage (Figure 9). Optimally, the device should not protrude >4–7 mm beyond the LAA ostium, and residual flow should be <5 mm by color Doppler with a compression grade of  8–20%, expressed in percent comparing the diameter of the implanted device with the unconstricted diameter indicated by the manufacturer. When optimal positioning is confirmed, the device is released. Rare device embolization after mobilization of the patient has been observed.

Following successful device deployment, the pericardium is evaluated for effusion.

### 4.3. Follow-Up Imaging

Postprocedural imaging aims to assess device position, peridevice residual flow in the LAA, and thrombus formation on the device.

**Figure 10:**
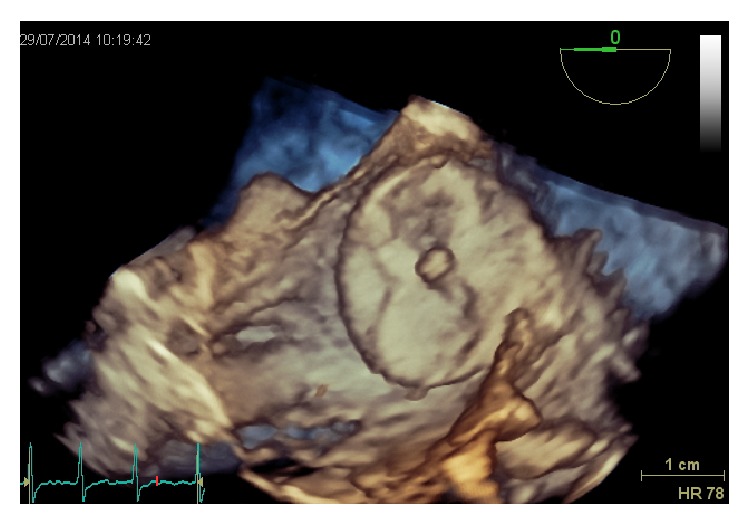
An Amplatzer Cardiac Plug six months after implant, with perfect sealing and endothelization.

TEE (Figure 10) and CT can both be used for this purpose.

In the PROTECT AF trial, serial TEE imaging was performed at 45 days, 6 months, and 1 year following implant [38].

Residual peridevice flow is common (41% at 45 days) in patients treated with the Watchman device. It is unclear if this could be related to possible thromboembolic events, since new thrombi may be formed in the distal LAA pouch [48]. Of note in the PROTECT AF trial, patients with a peridevice flow did not have a worse clinical outcome, regardless of the chosen antithrombotic therapy (warfarin, double antiplatelet agents, ASA).

With Amplatzer devices, as the disc of the device typically covers the entire LAA ostium (pacifier principle), residual peridevice leaks are less frequent.

### 4.4. Anticoagulation after Implantation

Postprocedural antithrombotic therapy with warfarin or dual antiplatelet drugs is recommended after implantation to avoid thrombus formation on the device until completion of endothelialization, provided there are no contraindications. For the Watchman device, the antithrombotic protocol of the PROTECT AF trial is adopted: 45 days after implantation warfarin was discontinued and substituted by ASA + clopidogrel if the TEE showed the absence of thrombi or a residual peridevice flow of <5 mm in width; clopidogrel was stopped if the 6 months TEE follow-up demonstrated no complications.

Usually, one antiplatelet agent is continued indefinitely, as most patients are elderly with evidence of atherosclerotic disease, although the bleeding risk must be considered.

The PREVAIL trial and ASAP study provided evidences for double antiplatelet therapy instead of VKA for the Watchman device.

Observational studies with the ACP followed a regimen of clopidogrel + ASA for 1 month and acetylsalicylic acid for 3 to 6 months [60], borrowing the antithrombotic protocol from the experience with the Amplatzer PFO Occluder. In patients who are treated with antiplatelets drugs, it is reasonable to perform an imaging test (TEE or CT scan) before clopidogrel termination and again if ASA cessation is planned.

Incomplete LAA occlusion could create, theoretically, a thrombus-containing pocket with a source of possible systemic embolization. Anyway, as mentioned above, small residual shunts (<5 mm) are usually considered irrelevant and may close spontaneously with time. When all patients with residual shunts are included, the stroke risk is no different compared with patients in whom the LAA is completely occluded regardless of antithrombotic therapy [48]. However, this remains a field open for debate.

## 5. Indications for Left Atrial Appendage Occlusion

PROTECT AF and PREVAIL randomized controlled trials were included in the recent ESC focused guidelines on stroke prevention in patients with atrial fibrillation. In fact, they suggest to use the CHA2DS2-VASc risk score** >**1 as the threshold value for considering LAA occlusion [16].

Individual risk-benefit evaluation is fundamental, bearing in mind that the use of OAC has a primary recommendation.

### 5.1. When Anticoagulation Is Not Possible

Patients with a high thromboembolic risk (CHA2DS2-VASc score of ≥2) but contraindication to systemic anticoagulation (e.g., history of intracranial or life threatening bleeding, coagulation disorders) represent the most accepted indication for LAA occlusion. In a survey of European centers, the most common indication for LAA closure was an absolute contraindication to OAC [61].

So far, no randomized trials targeting this specific group of patients are available; in fact, this is the result of several observational studies and registries. However, the significant bleeding risk of dual antiplatelet therapy, indicated for 1 or 6 months after implantation, has to be considered [62]. Generally, this is only for a short time, thus reducing the cumulative risk of major bleeding events.

In patients who cannot receive any antiplatelet agent, the Lariat technique can be considered.

### 5.2. When Oral Anticoagulation Is Possible

This is the only indication, as cited above, that is based on randomized controlled trials. Those patients, in whom OAC or NOAC are considered to pose an unacceptable bleeding risk (HAS-BLED ≥ 3), but with high stroke risk (CHA2DS2-VASc score of ≥ 2), should be considered for LAA occlusion.

The possibility of LAA occlusion should be discussed with the patient, remembering that anticoagulation currently remains the standard of therapy.

Patients should be elucidated about the possibility of therapy with NOAC, that, compared to OAC, have at least an equivalent and probably improved efficacy, with lower rate of intracranial and, for some, lower overall bleeding risk.

It should be emphasized that we do not have any direct data comparing NOAC with LAA occlusion.

Ultimately, the decision belongs to a well-informed patient in collaboration with the physician.

The HAS-BLED score does not characterize the bleeding risk in certain categories of patients (e.g., patients with cancer or chronic inflammatory bowel disease): to these, LAA occlusion may also be offered.

Another possible scenario in which LAA occlusion could be helpful is the setting of triple anticoagulant therapy due to coronary stent interventions in AF patients as it poses a significant rise in bleeding risk [63].

End-stage renal failure poses patients at a high stroke risk and high bleeding risk: LAA occlusion could be a debatable alternative, keeping in mind that all NOAC are contraindicated with creatinine clearance** <** 15 mL/min and warfarin could increase tissue calcification and enhanced atherosclerosis in this setting.

### 5.3. As a Complement to Anticoagulation

The combination of LAA occlusion and OAC is debated and occasionally performed in patients with embolic events despite the adequate antithrombotic therapy, provided that there are no other plausible causes (e.g., patients with mechanical prosthetic valves with evidence of thrombus in the LAA).

## 6. Conclusions

The LAA is considered the “most lethal human appendage” as it causes significant mortality and morbidity in AF patients. We have to learn more about its complex role in physiology and pathology.

However, LAA occlusion is becoming an interesting tool in reducing thromboembolic risk in certain categories of patients, as it has become a safe and effective procedure.

Indeed, we need new randomized prospective trials comparing LAA procedure with NOAC, as they have a safety profile better than old OAC.

Other interesting aspects that warrant investigation are the clinical significance of residual peridevice flow and the correct antithrombotic therapy after LAA closure.

In this way, we can shed light on thromboembolism management in AF, in order to improve our knowledge when choosing between different measures to reduce the risk of catastrophic strokes.

## Figures and Tables

**Table 1:** Results with the Watchman device from Meier et al. [16].

Trial	Patients	Patients device/ control	Comments	Average CHADS2 Score	Average CHA2DS2-VASc Score	Medical therapy	Efficacy events	Safety events	Successful implantation	Mean follow-up (months)	No warfarin	Primary efficacy event rate (per 100 patient-years)	Safety event rate
Pilot study	66	66/0	Nonrandomized cohort of patients undergoing Watchman implantation	1.8 ± 1.1		Warfarin plus ASA for 45 days and ASA for life	Death, stroke, systemic embolism, and major bleeding		88%	73 ± 25	91%	Actual stroke rate of 0.5%	4-device embolization

PROTECT AF	707	463/244 warfarin	Randomized noninferiority trial	2.2 ± 1.2	3.4	Warfarin plus ASA for 45 days, DAPT for 6 months, and ASA for life	Composite endpoint of stroke, cardiovascular death, and systemic embolism	Device embolization, major bleeding events, and pericardial effusion	88%	18 ± 10 43.4 ± 21.7	94%		

CAP registry	460	460/0	Nonrandomized registry of patients undergoing Watchman implantation	2.4 ± 1.2		Warfarin plus ASA for 45 days, DAPT for 6 months, and ASA for life	PROTECT AF protocol	PROTECT AF protocol	95%	25.4 ± 10.0	95%	2	

ASAP registry	150	150/0	Treat patients contraindicated for warfarin	2.8	4.4 ± 1.7	DAPT for 6 months and ASA for life	Stroke rate per 100 patient-years		95%		100%	2	

PREVAIL	407	269/138	Similar to PROTECT AF with revised inclusion criteria	2.6 ± 1.0		Similar to PROTECT AF	Stroke, embolism or unexplained death	Same as PROTECT AF within 7 days	95.1%		Modelled to 18 months; only 58 actually reached 18 months	1	4

**Table 2:** ACP registries in comparison with PROTECT AF.

In-hospital											Follow-up				
Registry	Patients	Mean age (year 5)	Mean CHADS2 score	Technical success	Stroke	Pericardial effusion conservative	Tamponade (drainage)	Device embolization	Death (all causes)	Total adverse events	Device embolization	Pericardial effusion	Thrombus on device	Stroke	Death
Italian	100			100/100	0		2/100	0	0	2/100					
Registry				100%			2%			2%					
Dual Centre	131			131/131	0	1/131	0	0	0	1/131					
Hamburg				100%		1%				0.8%					
Bern															
ACP EU Post	204	74 ± 9	2.6 ± 1.3	197/204	0		3/204	3	0	6/204	1	0	5/204		
Market				97%			1.5%			2.9%			2.4%		
Registry															
Spanish	35	75 ± 6	2.4 ± 1.3	34/35	0	0	0	0	0	0	0	0	5/35	1/35	3/35
Registry				97%									14%	3%	9%
Initial	143	74 ± 9	—	132/137	3/143	4/143	5/143	2/143	0	10/143					
European				96%	2.1%	3%	3.5%	1.4%		7%					
Experience															
Bern LAA	100	72 ± 10	2.5 ± 1.3	98/100	1/100	2/100	1/100	2/100	0	6/100					
Occlusion				98%		2%	1%	2%		6%					
Registry															
Initial Asian	20	68 ± 9	2.3 ± 1.3	19/20	0	0	0	0	0	∗	—	—	—	—	—
Experience				95%											
Canadian	52	74 ± 8	3 (2–4)	51/52	0	1/52	1/52	1/52	0	2/52	0	1/52	0	1/52	3/52
Registry				98%		2%	2%	2%		4%		2%		2%	6%
PROTECT AF	463	72 ± 9	2.2 ± 1.2	408/463	5/463	8/463	22/463	3/463	0	36/463	2/463	0		16/694	21/705
				88%	1%	1%	5%	1%		8%	0.4%			2.3%	3.0%

∗: Air embolism in right coronary artery, one esophageal injury during TOE.
